# Adapting and Validating a Patient Prompt List to Assist Localized Prostate Cancer Patients with Treatment Decision Making

**DOI:** 10.3390/healthcare12191981

**Published:** 2024-10-04

**Authors:** Levi Ross, Linda Collins, Florida Uzoaru, Michael A. Preston

**Affiliations:** 1 Department of Kinesiology and Health Sciences, Southeastern Louisiana University, Hammond, LA 70402, USA; 2 Department of Health and Human Sciences, Southeastern Louisiana University, Hammond, LA 70402, USA; lcollins@southeastern.edu (L.C.); florida.uzoaru@selu.edu (F.U.); 3 Department of Pharmacy Practice, Purdue University, West Lafayette, IN 47907, USA; mapreston@purdue.edu; 4 Center for Health Equity and Innovation, Purdue University, Indianapolis, IN 46202, USA

**Keywords:** patient prompt list, content validity, prostate cancer

## Abstract

Background/Objectives: Effective communication between patients and healthcare providers is essential for informed decision making in cancer care. Communication aids that can help prostate cancer patients optimize their involvement in treatment care planning are not widely used in the U.S. This research details the adaptation and validation process of a patient prompt list for localized prostate cancer patients undergoing treatment decisions. Methods: This process occurred in three steps: Step 1 involved gathering usable questions from the literature; in Step 2, we evaluated the tool’s content via expert (N = 6) feedback; and in Step 3, we assessed the usefulness of the prompt list with patients (N = 30). Results: Sixty percent of candidate questions (20/33) were retained for inclusion after achieving acceptable item-level content validity index (range: 0.83–1.00) and scale-level content validity index (S-CVI = 0.96) scores. The final 20 questions were classified into 5 domains (1: Treatment Options and Information, 2: Side Effects, Risks, and Recovery, 3: Emotional and Social Support, 4: Logistical and Practical Concerns, and 5: Future Health Considerations) through a card sorting exercise with a subset of patients (N = 3) and providers (N = 2). Most patients rated the prompt list as “useful or very useful” both to themselves (80%, N = 24) and to other men presenting with prostate cancer (83%, N = 25). Conclusions: The participatory process used to develop and validate the prompt list offers insights for the development of similar tools.

## 1. Introduction

Prostate cancer is the most common cancer among American men. In 2024, 299,010 men are expected to be diagnosed, and 32,250 will die from the disease [1]. Despite the high prevalence of the disease, decision making in treatment is fraught with challenges. Some of the well-documented clinical and patient-level factors that complicate treatment care planning for patients with localized disease (stage T1 or T2) include the availability of different therapeutic options (e.g., surgery, radiation, and active surveillance), the existence of treatment side effects [2], uncertainty in predicting who will experience known side effects [3], and differences in patients’ tolerance of outcomes that compromise quality of life [4].

The duration of a cancer office visit can vary, but studies have shown that the average time for an ambulatory prostate cancer consultation is 18.5 min (SE ± 0.7 min) [5]. During a visit involving treatment decision making, physicians need to communicate a vast amount of information, including diagnosis and treatment options [6]. In addition to answering patient questions, physicians must also use this time to provide emotional support and perform knowledge checks to assess patients’ comprehension of the information that is presented to them [7].

Even though treatment consultations last slightly longer than general appointments, many newly diagnosed patients leave cancer treatment decision making visits feeling that their concerns have not been adequately addressed [8]. Men who are unable to express themselves effectively during the time constraints of an emotionally charged treatment consultation can become demoralized, anxious, and confused and make decisions that are misaligned with their preferences or lifestyles [9]. Cancer prevention and control experts can improve the patient treatment decision making experience by helping men to optimize the time they spend with physicians.

Question prompt lists are low-tech, easy-to-use aids that can help patients develop communication plans for a clinical interaction [10]. These lists can be generic or disease-specific and include specific questions or focus on general content areas. In the field of oncology, prompt lists have been used with multiple disease foci (e.g., brain [11], breast [12,13], head and neck [14], and melanoma [15]) in several countries (e.g., Australia [10,14,15,16], Canada [17], France [18], India [19], Japan [20], and the US [21]) at different points of a patient’s illness (e.g., genetic testing [22], treatment [19], and palliative care [14,18]). Prompt list studies have shown that this communication intervention can enhance patient engagement and improve the exchange of information, which in turn helps patients to find better solutions to their health problems. Despite the documented benefits of this form of communication assistance, there are a lack of validated prompt lists that have been designed for use with prostate cancer patients in the U.S.

The treatment decision making experience in the U.S. differs from other countries in many ways. For example, in nations with universal public health insurance (e.g., Canada, UK), treatment decisions are less influenced by out-of-pocket costs than in the U.S. [23]. The treatment culture for low-risk cancers tends to be more aggressive in the U.S. compared to other countries in which there is greater trust in long-term monitoring without immediate intervention [24]. The U.S. also has a higher number of specialized treatment centers that can give patients easier access to advanced therapies or different treatment options [25]. U.S. cancer patients also have greater access to information through online resources and advocacy groups than in countries where access to information can be more controlled or limited. Given these and other reasons not mentioned, a prompt list developed in other countries may fail to resonate with U.S. patients and providers without sociocultural tailoring.

This study sought to improve the quality of communication between prostate cancer patients and healthcare providers by adapting and validating a patient prompt list for use with localized prostate cancer patients who are facing treatment decisions. The validation process included (1) conducting a literature review of existing questions to generate a draft prompt list, (2) customizing the prompt list based on expert feedback [content validity indices], and (3) assessing the prompt list’s usefulness based on patient evaluations.

## 2. Materials and Methods

### 2.1. Participants

The data presented here are part of a larger study on quality of life among prostate cancer patients. The larger study collected participant observations, ethnographic interviews, Delphi panels, and survey research data from over 100 prostate cancer patients, their significant others, and treating healthcare providers. The current research analyzed a subset of unpublished Delphi panel data with healthcare providers and survey data from patients.

A total of 6 healthcare providers specializing in prostate cancer care and 30 patients were purposively recruited to participate at a teaching hospital and through community outlets. The provider group included 2 attending urologists, 2 urology fellows, an oncology nurse, and an oncology social worker. Providers were eligible to participate if they were employed at the recruiting hospital; licensed in their field; and had a minimum of two years experience of providing care to prostate cancer patients. Patients were eligible to participate if they self-identified as white or African American; could read and write English; and were treated for or monitoring localized prostate cancers (stage T1 or T2) within 5 years of recruitment. All healthcare providers were recruited through targeted outreach to department and tumor board meetings by the study PI. Patients were recruited via electronic medical records, word-of-mouth, posted flyers, and advertisements in local newspapers.

#### Ethical Approval and Informed Consent

All healthcare providers and patients provided written informed consent to participate prior to enrollment. All study-related information that existed in paper form was kept in a locked filed cabinet in the PI’s office. All study data that existed electronically were kept in a password-protected database on the PI’s work computer. The study was approved by the Institutional Review Boards at Roswell Park Cancer Institute (I-198711) and Georgia Southern University (H-14043).

### 2.2. Procedures

#### 2.2.1. Literature Review and Selection of Patient Prompt List Questions

A comprehensive literature review was conducted to identify candidate questions that could be included on the treatment decision making prompt list for men with early stage disease. Members of the research team searched the PubMed database using the search terms prostate cancer, question prompt list, patient prompt list, and treatment information needs. This search returned no adaptable published prompt lists [26]. However, several articles included questions that were deemed potentially useful for creating a new prompt list [27,28,29,30,31,32]. After hard or electronic copies of the selected articles were obtained, the candidate questions were abstracted and catalogued in an Excel spreadsheet for healthcare providers to review.

#### 2.2.2. Validation of the Patient Prompt List

Each healthcare provider was asked to independently review the list of 27 candidate questions and provide his/her opinion on each question’s relevance to a patient’s encounter during a treatment decision making consultation. The goal was to create a streamlined list that could be easily incorporated into a prostate cancer treatment consultation without overwhelming patients [33]. With this goal in mind, the experts were asked to prioritize questions that were essential and eliminate any that were redundant or less relevant. Each item was rated on a 4-point scale, with response options being 1 = not at all relevant, 2 = somewhat relevant, 3 = quite relevant, and 4 = highly relevant. The scores produced from experts’ ratings allowed us to produce two content validity indices.

The item-level content validity index (I-CVI) is an item-level validity measure. This index represents the proportion of individual experts who rate an item as quite or highly relevant. With this index, expert ratings of 1 (not at all relevant) or 2 (somewhat relevant) are considered not relevant, and ratings of 3 (quite relevant) or 4 (very relevant) are relevant. The I-CVI ranges from 0–1, with values ≥ 0.80 considered acceptable [34]. The CVI was calculated using the formula below.

I-CVI = Number of experts rating the item as 3 or 4/(Total number of experts)

The scale-level content validity index (S-CVI) is a scale-level index that measures the collective relevance of all items included in the prompt list. The S-CVI is computed by averaging the I-CVIs for all individual prompt list questions. The S-CVI ranges from 0–1, and values ≥ 0.90 are considered acceptable [35]. The S-CVI is calculated using the following formula:

S-CVI = (∑I-CVI)/(Number of items)

Questions were retained on the prompt list if they met the I-CVI and S-CVI thresholds of acceptability mentioned above. Questions that did not meet the thresholds of acceptability for the I-CVI were revised and re-evaluated if a revision recommendation was made by a healthcare expert. The revised questions were then subjected to a second round of evaluation to determine if the changes improved their I-CVI scores. This iterative revision process helped to ensure that potentially valuable questions were not prematurely excluded. Questions that did not meet the retention cut-offs and did not receive any suggestions for revisions were eliminated from the list. Experts were also instructed to suggest items for inclusion on the prompt list that were not included from the literature review. This final step was included to ensure the prompt list was comprehensive and reflective of current clinical practice. All suggested questions were subject to the same evaluation process as the original questions, and their relevance was assessed via the I-CVI and S-CVI.

#### 2.2.3. Patient Prompt List Usefulness Evaluation

After the prompt list was finalized, 30 patients were given a paper version of the tool to assess its applicability from a user’s perspective. Each patient was asked to provide feedback on three areas: (a) the importance of each question on the list, (b) the perceived usefulness of the prompt list to them personally, and (c) the perceived usefulness of the prompt list for other men diagnosed with prostate cancer.

The importance of the list was assessed with the question, “Think back to when you were diagnosed with prostate cancer. How important was it for you learn about ______ to make the best treatment decision?” Response options included 1 = not at all important, 2 = a little important, 3 = somewhat important, and 4 = very important. This item was designed to determine if the questions included on the prompt list addressed the core concerns that patients typically have when weighing their treatment options.

Perceived helpfulness to oneself was evaluated with the question, “Now that you have reviewed the prompt list in its entirety, how helpful would it had been for you to receive this list when you were first diagnosed?” Response options included 1 = not at all helpful, 2 = a little helpful, 3 = helpful, and 4 = very helpful. This feedback helped the research team understand how well the prompt list addressed common uncertainties or gaps in knowledge that men might face when deciding between treatment options.

Perceived helpfulness to others was evaluated with the question, “How helpful do you think this prompt list would be to other men who are newly diagnosed with prostate cancer?” Response options included: 1 = Not at all helpful, 2 = A little helpful, 3 = Helpful, and 4 = Very helpful. This question sought to capture participants’ perceptions of how the prompt list could benefit men facing prostate cancer for the first time.

#### 2.2.4. Questions Grouping into Thematic Domains

Following the content validation and patient evaluation process, a subset of participants (3 patients, 2 health experts) were asked to participate in an individually moderated card sorting activity to organize the final prompt list questions into meaningful categories. Each participant met independently with a research staff member and was provided a packet containing each final prompt list question listed separately on an index card. Participants were instructed to sort all of the cards into at least two groups based on their perceptions of similarity. Participants could have provided labels for their card groupings, but labeling was not a requirement. No predefined categories were given or suggested to participants to allow for natural clustering.

The research team uploaded each participants’ grouped questions into NVivo 12™, which is a piece of qualitative data management and analysis software. The cluster analysis function was used to determine similarities between all participant produced groupings (nodes). Vertical dendrograms were produced to allow the research team to visually inspect common themes (nodes) across participants’ groupings using the ‘questions coding similarities’ clustering option. This qualitative analysis focused on the frequency with which prompt list questions were grouped together. The research team provided labels for each domain after agreement on the questions’ clustering was obtained.

### 2.3. Data Analysis

Both content validity indices were calculated in Microsoft Excel^®^ (Excel 2019). Univariate statistics were produced in the Statistical Package for the Social Sciences (SPSS 25, IBM Corp. Armonk, NY, USA) to describe participants’ evaluations of the prompt list importance and its perceived helpfulness. NVivo 12™ was used to group prompt list questions into thematic domains.

## 3. Results

### 3.1. Content Validity

The healthcare experts’ initial judgment of the prompt list questions is included in Table 1. After the first round of reviews, 14 items met the threshold for retention (Q1, Q3, Q5, Q7, Q8, Q10, Q11, Q12, Q13, Q16, Q22), and 13 items were recommended for deletion (Q4, Q9, Q14, Q15, Q18, Q19, Q20, Q21, Q24, Q25, Q26, Q27) because of redundancy or inappropriateness. The panel of experts thought that six questions were missing from the initial list that would be relevant to localized patients’ treatment decision making experiences (Q28, Q29, Q30, Q31, Q32, Q33) in the US. The S-CVI for the first round of prompt list questions fell below the 0.90 threshold of acceptability (S-CVI = 0.74).

The healthcare experts’ final version of the prompt list is shown in Table 2. The questions listed on the revision included the 14 items that were retained from the original review and the 6 items that were added based on expert suggestions. The I-CVIs for the questions on the revised prompt list ranged from 0.83 to 1.00. The S-CVI for the revised list of questions increased to 0.96.

### 3.2. Patients’ Ratings of the Importance of Prompt List Questions

**Figure 1:**
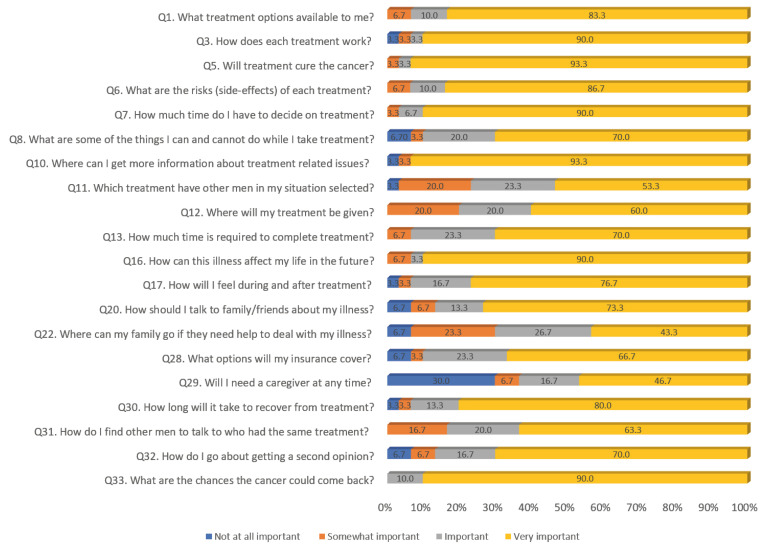
Patients’ evaluations of the importance of prompt list questions.

Patients’ evaluations of the usefulness of the finalized prompt list are included in Figure 1. The proportions of men who thought the prompt list items were “important or strongly important” ranged from 63.4% for “Would I need a caregiver at any time?” to 100% for “What are the chances the cancer could come back after treatment?”

### 3.3. Usefulness of the Patient Prompt List to Oneself and Others 

**Figure 2:**
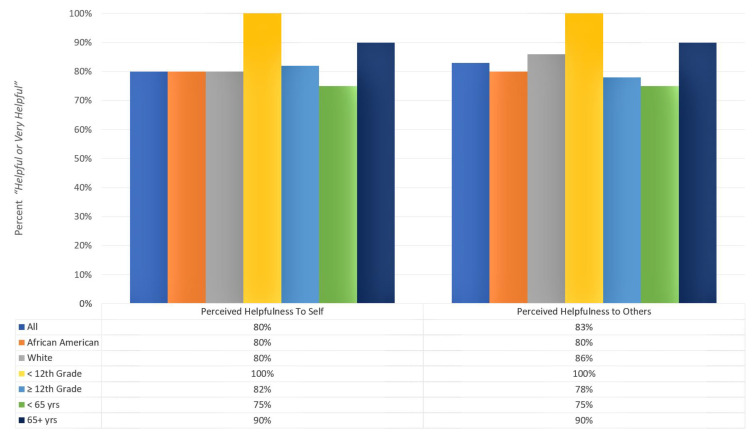
Patients’ judgments of the helpfulness of the prompt list by demographic characteristics.

Patients’ judgements of the helpfulness of the 20-item prompt list, both to themselves and others are included in Figure 2. Most men in the sample viewed the prompt list as being “helpful or very helpful” to themselves (80%, N = 24) and “helpful or very helpful” to other men presenting with prostate cancer (83%, N = 25).

Similar positive evaluations were made regarding the prompt list being “helpful or very helpful” to oneself and others across demographic characteristics. Equal proportions of white people (80%, N = 12/15) and African Americans (80%, N = 12/15) rated the prompt list as “helpful or very helpful” to themselves. This positive evaluation was consistent across different education levels (100% < high school vs. 82.0% ≥ 12th grade) and age groups (75.0% < 65 years vs. 80.0% ≥ 65 years).

The majority of men in our racial (86.0% whites vs. 80.0% African American), age (75.0% < 65 years vs. 90.0% ≥ 65 years) and education groups (100% < high school vs. 78.0% ≥ 12th grade) also rated the prompt list as “helpful or very helpful” to other men.

### 3.4. Prompt List Questions Groupings by Thematic Domains

**Figure 3:**
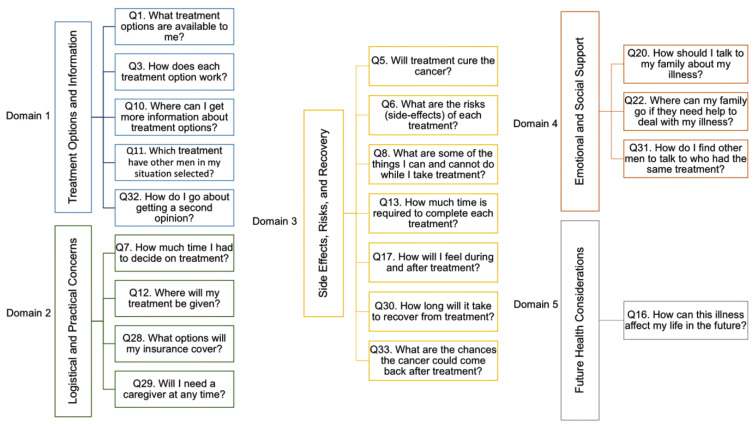
Prompt list questions grouped by thematic domains.

The card sorting activity resulted in the final 20 prompt list questions being organized into 5 interrelated domains. A brief description of each domain is provided below. A visual representation of questions grouped by domains is included in Figure 3.

Domain 1: Treatment Options and Information—This domain includes questions (Q1, Q3 Q10, Q11, Q12) that help patients gain a thorough understanding of the various treatment options available to them. Questions in this category focus on the mechanics and outcomes of each treatment and ways to gather more information. Examples include “What treatment options are available to me?” and “How does each treatment option work?” These questions are critical to treatment decision making because they help patients become well informed about their choices.

Domain 2: Side Effects, Risks, and Recovery—This domain (Q5, Q6, Q8, Q13, Q17, Q30, Q33) addresses the potential side effects and risks associated with each treatment option and the treatment recovery process. Questions such as “What are the risks (side effects) of each treatment?” and “How long will it take to recover from treatment?” help patients anticipate and prepare for the physical and emotional challenges they may face during and after treatment. These types of questions help men set realistic expectations and prepare for the practical aspects of their treatment journey.

Domain 3: Emotional and Social Support—This domain (Q20, Q22, Q31) focuses on the emotional and social aspects of coping with prostate cancer. Questions like “How should I talk to family/friends about my illness?” and “How do I find other men to talk to who had the same treatment?” are included in this domain. These questions underscore the importance of emotional well-being and social support as essential components of a holistic cancer care treatment plan.

Domain 4: Logistical and Practical Concerns—This domain covers practical issues that patients need to consider, such as insurance coverage, the need for a caregiver, and the logistics of receiving treatment. “What options will my insurance cover?” and “Will I need a caregiver at any time?” are key questions in this category. Addressing these concerns will help patients plan effectively for the financial and practical aspects of their treatment.

Domain 5: Future Health Considerations—This domain includes a single question that encourages patients to consider the long-term implications of their illness and treatment. The key question in this domain is “What are the chances the cancer could come back after treatment?”

## 4. Discussion

This study adapted and validated questions from the literature into a patient prompt list for men with localized prostate cancer to assist them with treatment decision making. The final 20-item prompt list achieved high content validity after a rigorous validation process with survivors and providers. Incorporating prostate cancer survivors into a retrospective analysis of the prompt list added value to the study because their opinions were based on hindsight. Having already been through the treatment decision making process, these men know what information they needed and how well this tool would have helped them fulfill their information deficiencies.

One of the strengths of the prompt list is its organization into five thematic areas. These patient- and physician-derived categories allows patients to engage with the prompt list in an individualized, yet structured manner. For instance, a patient who is primarily concerned about side effects can focus on that section first before moving to other areas. Conversely, a patient who is more concerned with long-term outcomes can navigate to those questions first before moving on the remaining areas. This personalization feature can help patients feel a sense of control, as it allows them to prioritize the issues that matter most to them, without feeling overwhelmed by information that may not be immediately relevant.

When placed in the context of ongoing efforts to enhance cancer care, our findings underscore the importance of rigorous content validation processes when developing patient-centered tools for new populations. The significant improvement in the scale-level content validity index (S-CVI) from 0.74 to 0.96 after expert feedback highlights the value of using a participatory approach. By leveraging the diverse insights of professionals across different specialties, the prompt list was also refined to better align with the complexities of the holistic patient experience.

One unexpected result from the study was the recommendation to delete 13 of the original 27 questions due to perceived redundancy. This was surprising because those questions were selected from a comprehensive literature review [27,28,29,30,31,32]. After placing the prompt list questions into domains, we saw there was overlapping content with some of the items that were being considered for inclusion. By removing the overlapping questions, the prompt list was streamlined for clarity and usability. We achieved our goal of developing a comprehensive yet brief tool to avoid overwhelming patients during a challenging time in their cancer journey. The final 20 items were designed to serve as gateway conversation starters to empower patients and open up dialogue. None of the five prompt list domains were lost as a result of the streamlining process.

### 4.1. Limitations

Despite its positive results, this study has some limitations that must be acknowledged. The sample size was relatively small and geographically limited. These two issues may limit the generalizability of our findings to broader populations (e.g., other racial, socioeconomic, or geographic groups). Future studies should include a larger and more diverse cohort to validate the tool more comprehensively. This can be achieved by partnering with multiple research sites across regions or using online platforms to enhance the recruitment pool. Since this study is an initial exploratory validation rather than a conclusive validation applicable to all settings, these issues were not considered in this research.

This study also relied on retrospective patient evaluations, which could introduce recall bias. With the passage of time, patients may not have accurately recalled their decision making experience, which could have led them to underestimate or overestimate the tool’s usefulness. Future research would benefit from real-time assessments with patients as they use the prompt list during actual treatment consultations. These additional studies could be longitudinal and track patient outcomes over several months to assess the long-term impact of the tool.

We did not measure the actual impact of prompt list use on measurable clinical outcomes, such as questions asked, decision making quality, or satisfaction with the clinical encounter. Future studies should incorporate standardized health and quality-of-life measures, such as patient-reported outcome measures (PROMs) or the SF-36, to enhance the prompt list’s credibility and demonstrate additional practical benefits. Measuring and demonstrating improvement in clinical outcomes can enhance the prompt list’s reputation as a useful resource for promoting patient-centered care. 

### 4.2. Future Directions

Broadly disseminating the prompt list is critical to maximizing its use. One of the strengths of the tool is its ability to be adapted into other accessible formats. In telehealth settings, the prompt list can be included as a pre-appointment form to help patients select questions they want to discuss during their upcoming appointment. The list of questions can also be displayed on the computer screen while patients are in the virtual waiting room waiting to be connected to their providers. The prompt list could be included as an intake form in electronic health records to trigger physicians to address specific concerns that are identified by patients. In mobile applications and electronic devices, providers can send push notifications to remind patients to review the prompt list before their office visits. These future research projects should take place in the U.S. and non-U.S. settings.

## 5. Conclusions

This research lays the groundwork for future studies validating prompt lists across diverse populations and healthcare settings. It will also create opportunities for integrating this form of communication assistance into digital health platforms.

## Figures and Tables

**Table 1:** Health experts’ initial judgement of the relevancy of candidate patient prompt list questions.

Candidate Prompt List Questions	S-CVI (Scale)	Item-Level Agreement	Decision
Q1. What treatment options are available to me?	0.75	1.00	Retain
Q2. Which treatment does he/she recommend for me?	0.75	0.67	Delete
Q3. How does each treatment option work?	0.75	1.00	Retain
Q4. What are my chances of dying from this illness with each treatment?	0.75	0.33	Delete—Covered under Q6 or Q16
Q5. Will treatment cure the cancer?	0.75	1.00	Retain
Q6. What are the risks (side effects) of each treatment?	0.75	1.00	Retain
Q7. How much time do I have to decide on treatment?	0.75	1.00	Retain
Q8. What are some of the things I can and cannot do while I take treatment?	0.75	1.00	Retain
Q9. What caused my problem?	0.75	0.50	Delete
Q10. Where can I get more information about treatment-related issues?	0.75	1.00	Retain
Q11. Which treatment have other men in my situation selected?	0.75	1.00	Retain
Q12. Where will my treatment be given?	0.75	1.00	Retain
Q13. How much time is required to complete each treatment?	0.75	1.00	Retain
Q14. What are the chances my illness might spread with no treatment right away?	0.75	0.50	Delete—Covered under Q5
Q15. What treatment can I receive later if my first treatment choice is unsuccessful?	0.75	0.50	Delete—Covered under Q3
Q16. How can this illness affect my life in the future?	0.75	0.83	Retain
Q17. How will I feel during and after treatment?	0.75	1.00	Retain
Q18. Who should I call if I have any concerns during treatment?	0.75	0.33	Delete—Covered under Q12
Q19. What side effects should I report to the doctor/nurse?	0.75	0.67	Delete—Covered under Q6
Q20. How should I talk to family and friends about my illness?	0.75	0.83	Retain
Q21. Can I continue with my usual activities (e.g., hobbies)?	0.75	0.67	Delete—Covered under Q8
Q22. Where can my family go if they need help to deal with my illness?	0.75	0.83	Retain
Q23. Who do I talk to about alternative treatments?	0.75	0.50	Delete—Covered under Q10
Q24. What do the results of blood tests mean?	0.75	0.67	Delete—Covered under Q5
Q25. Will there be changes in the usual things I can do with and for my family?	0.75	0.67	Delete—Covered under Q8
Q26. Are there groups available to talk to other people who had prostate cancer?	0.75	0.50	Delete—Will cover under recommended addition
Q27. Are there ways to prevent/ease treatment side effects?	0.75	0.67	Delete—Covered under Q6

**Table 2:** Health experts’ second judgement of the relevancy of the candidate patient prompt list questions.

Candidate Prompt List Questions	S-CVI (Scale)	Item-Level Agreement	Decision
Q1. What treatment options are available to me?	0.96	1.00	Retain
Q3. How does each treatment option work?	0.96	1.00	Retain
Q5. Will treatment cure the cancer?	0.96	1.00	Retain
Q6. What are the risks (side effects) of each treatment?	0.96	1.00	Retain
Q7. How much time do I have to decide on treatment?	0.96	1.00	Retain
Q8. What are some of the things I can and cannot do while I take treatment?	0.96	1.00	Retain
Q10. Where can I get more information about treatment-related issues?	0.96	1.00	Retain
Q11. Which treatment have other men in my situation selected?	0.96	1.00	Retain
Q12. Where will my treatment be given?	0.96	1.00	Retain
Q13. How much time is required to complete each treatment?	0.96	1.00	Retain
Q16. How can this illness affect my life in the future?	0.96	0.83	Retain
Q17. How will I feel during and after treatment?	0.96	1.00	Retain
Q20. How should I talk to family and friends about my illness?	0.96	0.83	Retain
Q22. Where can my family go if they need help to deal with my illness?	0.96	0.83	Retain
Q28. What options will my insurance cover?	0.96	1.00	Retain
Q29. Will I need a caregiver at any time?	0.96	1.00	Retain
Q30. How long will it take to recover from treatment?	0.96	1.00	Retain
Q31. How do I find other men to talk to who had the same treatment?	0.96	1.00	Retain
Q32. How do I go about getting a second opinion?	0.96	1.00	Retain
Q33. What are the chances the cancer could come back after treatment?	0.96	1.00	Retain

## Data Availability

The dataset used in this study is available from the corresponding author on reasonable request.
